# Associations between sex differences, eating disorder behaviors, physical and mental health, and self-harm among Chinese adolescents

**DOI:** 10.1186/s40337-023-00754-7

**Published:** 2023-02-27

**Authors:** Yuanyuan Wang, Zhihao Ma, Su Lu, Zhizhou Duan, Amanda Wilson, Yinwei Jia, Yong Yang, Runsen Chen

**Affiliations:** 1 Key Laboratory of Brain, Cognition and Education Sciences, Ministry of Education, South China Normal University, Guangzhou, China; 2 Guangdong Key Laboratory of Mental Health and Cognitive Science, School of Psychology, Center for Studies of Psychological Application, South China Normal University, Guangzhou, China; 3 Computational Communication Collaboratory, School of Journalism and Communication, Nanjing University, Nanjing, 210023 China; 4 Department of Psychology, Faculty of Health and Life Sciences, De Montfort University, Leicester, UK; 5 Preventive Health Service, Jiangxi Provincial People’s Hospital, The First Affiliated Hospital of Nanchang Medical College, 152 Aiguo Road, Nanchang, Jiangxi China; 6 Suzhou Guangji Hospital, Affiliated Guangji Hospital of Soochow University, Soochow University, Suzhou, China; 7 Vanke School of Public Health, Tsinghua University, No.30, Shuangqing Road, Haidian District, Beijing, China; 8 Institute for Healthy China, Tsinghua University, Beijing, China

**Keywords:** Eating disorders, Adolescents, China

## Abstract

**Background and aim:**

Eating Disorders (ED) result in impaired well-being, but there exist an insufficient number of studies that have focused on the influence of sex and sexual orientation disparities within ED behaviors. Thus, we aimed to investigate ED behaviors among male and female adolescents with different sexual orientations in a school sample to understand prevalence and correlates of different ED behaviors.

**Method:**

Data was analysed from 11,440 Chinese school adolescents with a mean age of 14.74 years (*SD* = 1.46). Reported data was gathered on sociodemographic information including sexual orientation, ED behaviors, health factors (reported health, cognitive function), mental health factors (depression, anxiety, suicidal ideation, non-suicidal self-injurious behavior), and social functioning (school bully victimization, and school bully perpetration). Logistic regression models were used to estimate the associations with ED behaviors, using the heterosexual orientation as the reference group as they are the majority.

**Results:**

Compared to female adolescents, male adolescents reported lower anxiety symptoms (*t* = − 12.39, *p* < 0.001, Cohen’s *d* = − 0.233), were more likely to be the perpetrator of school bullying (χ^2^ = 190.61, *p* < 0.001, φ = 0.129), and reported a lower likelihood of taking dietary restriction (χ^2^ = 290.08, *p* < 0.001, φ = 0.160). Overall, the prevalence of dietary restriction presented sex disparities. Adolescents who reported no sexual attraction were less likely to engage in ED behaviors. Using heterosexual orientation as the reference group, the group who reported no sexual attraction was associated with lower risk in dietary restriction and purging in both male and female adolescents. Using the heterosexual orientation as the reference group, female sexual minority groups were at high risk of ED behaviors, with bisexual orientation and gay/lesbian orientation having a higher likelihood of engaging in objective binge eating.

**Conclusions:**

The results revealed significant sex and sexual orientation differences of ED behaviors. The study suggests that adolescents is a period of sexuality development and could be critical for understanding adolescents’ eating behaviors. It is important to guide adolescents to healthy eating during their development and considerations should be made by clinicians when creating interventions for ED behaviors among the different sex and sexual orientation groups.

**Supplementary Information:**

The online version contains supplementary material available at 10.1186/s40337-023-00754-7.

## Summary

Poor reported health status, cognitive deficits, suicidal ideation, self-harm, mood disorders, and school bullying experiences were associated with ED behaviors among adolescents. Also, female sexual minority groups had a stronger association with ED behaviors. We recommended to implement tailored prevention strategies that are different for each of the adolescent groups studied, and that this needs to be specific to the different ED behaviors discussed.

## Introduction

Eating Disorders (ED) are psychiatric disorders characterised by pathological eating behaviors, which can lead to impaired physical health, psychosocial functions, and medical complications [68]. ED such as objective binge eating or extreme dietary restriction have been significantly associated with functional impairment in both work and life due to the impact that ED’s can have on mental and physical health [45, 64]. The prevalence of ED has increased in the recent half century, and researchers have recommended that eating habits should be routinely assessed as a part of general health assessments [68]. Adolescence is a period of body development, that results in adolescents being vulnerable to EDs, and the peak of onset is 15–19 years of age, which has arguably been linked to the onset of puberty’s hormonal and weight related changes [53]. In a longitudinal study over 30-years, sex differences were found in the psychological impact of the onset of puberty relative to peers, where late onset puberty was associated with abnormal eating attitudes and behavior among adolescent males but a protective factor for females; likewise earlier onset puberty was an associated factor for females, but there was no significant association for males [31]. There are also sex differences in current research looking at 14–19-year-olds that suggests being a victim of bullying and reported distress are two specific associations for developing ED during adolescence, with sex differences being reported where 19.4% of males and 44.6% of females met the threshold for ED, with adolescent females reporting worse peer relationships than males [13].

### Sex and ED behaviors

It is well-documented that there are sex differences in ED behaviors. Research has shown that males are more likely to perceive overeating, while females are more likely to perceive loss of control while eating [65]. Previous research in Australia suggests that when compared to females, objective binge eating has a greater impairment on males’ mental health, and that weight concerns have a greater impairment on females’ mental health [44]. Neumark-Sztainer and colleagues [49] further found that concerns about weight, weight related teasing, and dieting predicted ED behaviors in female adolescents, and concern over weight and weight control behaviors predicted these behaviors in male adolescents. The weight related concerns vary in males and females, where males are socialized to value muscularity and female are socialized to prefer a slim figure [68].

### Sexual orientation and ED behaviors

Besides the sex differences, research also indicates that there are eating behavior disparities among different sexual orientation groups [7]. Limited research has focused on different ED behaviors when considering sexual orientation, particularly for adolescent males and females, as sexual minority youth also are at a greater risk of EDs [2, 47]. A literature review does suggest that adolescents who are Lesbian, Gay, or Bisexual (LGB) are more prone to eating disorders than heterosexual adolescents, with the literature on lesbian adolescents being inconsistent in their findings. However, overall individual factors are considered related to the Minority Stress Theory [51]. In the Minority Stress Theory LGB populations perceive stressors including distal stress, such as discrimination and victimization, proximal stress, such as internalised homophobic stigma, and disclosure stress, such as the stress of concealment of sexual orientation, as well as social violence and victimization [51]. In an expansion of the theory researchers indicate that the ED behaviors reported by sexual minorities could be perpetuated by minority stress and discrimination [47, 51]. Research on ED among the LGB population has shown that there are more ED behaviors among gay males when compared to heterosexual males [1]. Compared to heterosexual counterparts, sexual minority youth perceive ED behaviors more frequently and they have a significantly higher prevalence of ED than heterosexuals [47, 77]. A recent meta-analysis revealed that compared to heterosexual females, a higher number ED behaviors were found among sexual minority female, with higher occurrences of binge eating and purging specifically [43]. Studies further show that sexual orientation could modify related ED behavior in male and females [7, 8, 21]. Bisexual female and gay males are found to have significantly higher body weight dissatisfaction than heterosexual participants, lesbians, and bisexual males [42]. Thus, those disparities in body weight dissatisfaction may consequently lead to disparities in ED behaviors among different sexual orientation groups. In addition, research has indicated that sexual orientation discrimination was related to similar or increased ED in lesbian youth, when compared to heterosexual females, as they perceive less familial social support, which is related to negative affect and social anxiety [40]. The research on sexuality and ED behaviors factor in that identifying as lesbian or a bisexual females may affect the eating behaviors of adolescents and young adults. For example, research suggests that lesbian or bisexual females face the same pressures to conform to heteronormative beauty standards such as thin and curvaceous, which are seen as more desirable to certain sexual partners and have higher rates of body satisfaction [58]. In contrast, when compared with heterosexual females, lesbians are considered significantly more likely to be overweight [3, 66]. These socio-cultural standards of sexual attraction and the reality of their body related features could impact on eating behaviors among different sexual orientations, however further research is needed before any conclusion can be made. It is also important to consider the negative social construction that comes with being a sexual minority, in concordance with the Chinese traditional cultural values [20, 74, 75], which could affect the bodily perception of young sexual minority people and consequently lead to their distorted perceptions of body shape resulting in ED behaviors.

#### Other variables associated with ED behaviors

Beside the disparities from sex and sexual orientation, there are variables proposed in previous studies that are closely associated with ED behaviors. Specifically, suicidal ideation and non-suicidal self-harm, reported cognitive deficits, anxiety symptoms, depressive symptoms, health status, school bullying experiences, and culture. These variables will be discussed in turn.

A previous meta-analysis showed that compared with the general population, people with anorexic tendencies are eighteen times more likely to die from suicide, and five times more likely to die prematurely from any other cause [26]. Thus, research on Anorexia Nervosa related concepts, such as dietary restriction, in adolescents is necessary. Furthermore, in a regression model study, with 82 adolescents, binge eating did not predict suicide where restrictive eating did [73]. It has been further suggested there is a high comorbidity of non-suicidal self-harm and ED behaviors [11], however the literature on adolescents predominantly focuses on adolescent females. In a sample of 47 adolescent females, who were admitted to hospital for Anorexia Nervosa, self-harm was found in 47% of the young females [28]. In contrast, a study of 189 female adolescent inpatients, showed a higher tendency of non-suicidal self-harm in the binge-purge group when compared to the restrictive eating group, with various personality factors influencing the prevalence [4]. The research in this area is still in its infancy with the protocol recently published for a mixed methods Self-Harm in Eating Disorders (SHINE) study [30], suggesting that understanding the risk of non-suicidal self-harm in sexual minority adolescent populations is also required.

Cognitive deficits, or mental impairments like an attention disorder, have also been linked to ED, with research suggesting that having a cognitive deficit can increase the risk of developing an ED [32]. This predisposition is also found in other studies, with a study of high-risk versus low-risk adolescents with ED finding that the high-risk group had deficits, specifically those with a higher risk of bulimia being more impulsive and having more theory of mind deficits when compared to those with anorexia [48]. Overall though there is a paucity of research on cognitive deficits and ED, but it is widely accepted the two concepts are related. The opposite is found when looking at common comorbid psychiatric problems, such as anxiety and depressive symptoms and ED, where there is a dearth of research. In a longitudinal study of adolescent boys and girls, having anxiety symptoms as a preadolescent can predict ED and the longitudinal effects of anxiety and ED can persist for 15 years after follow-up [70]. Not only can anxiety symptoms predict ED in adolescents but so can depressive symptoms, where being a female child predicts anorexia symptoms, and no known factors predicted the development of bulimia [5]. In another longitudinal study spanning a 15 year follow up period, depressive symptoms were predicted by dissatisfaction with one’s body, including ED outcomes like dieting, unhealthy weight control, and binge eating, particularly amongst female adolescents [54]. Similar to anxiety and depressive symptoms, the Health Questionnaire Short Form-36 has been widely studied in adolescents with ED, and is the most common measure of what we have referred to as health status in those with ED [15]. Though there is a dearth of research on anxiety and depressive symptoms and research using the health status survey, this has yet to be explored within sexual minority adolescents. The same is found with school bullying research, while it is concluded in a systematic review that bullying does predict ED [14], this has yet to be understood in sexual minority adolescents. Similar to the West, Chinese culture promotes fitness and shames obesity, with obesity signifying someone is unhealthy, ugly, clumsy, and/or stupid, irrespective of sex [34], which is fatphobic and highly stigmatizing. Though again the Chinese cultural context lacks explanation in most papers on Chinese adolescents with ED.

This study therefore aimed to explore the ED behaviors in adolescents and aimed to investigate the associated factors and health burden in a Chinese adolescent population, which could be used to inform future clinical interventions.

## Method

### Procedures and sample

Data for this study was cross-sectional and collected in Suzhou, China between June 2019 and July 2019 [75]. The participants were recruited in eighteen local secondary schools (grades 7–11). School teachers aided in the recruitment of participants. It was made clear to potential participants that participation was voluntary and that there were no adverse consequences for declining to participate or if later they withdrew from the study. The Ethics Committee of Suzhou Guangji Hospital approved the study protocol. A total of 12,354 adolescents completed the survey, with a response rate of 83.2%. All eligible samples (*N* = 11,440) provided their birth-assigned sex (the biological sex), gender identity, and what sex they were sexually attracted to [76]. A total of 914 students were excluded from analysis including: 227 students who did not provide a valid response on age (missing, below 10 years, and above 20 years were treated as invalid), 234 students who did not further provided their reported sex, and 453 students who could not further be identified as a certain type of sexual orientation.

### Measures

Socio-demographic information included: age, birth assigned sex, residence, whether they had any siblings, grade, and school residential status.

### Sexual orientation

The student’s sexual orientation was measured by two questions: “What is your reported sex (choosing from male or female)?” and “which sex are you sexually attracted to (choosing from male, female, both, or none)?”. Those male adolescents who were attracted to male were identified as gay adolescents; those female adolescents who were attracted to female were identified as lesbian adolescents; those who were attracted to opposite sex were identified as heterosexual adolescents; those who were attracted to both males and females were identified as bisexual adolescents; and those who were attracted to none were identified as reporting no sexual attraction. The measurement of sexual orientation was also adopted based on previous studies [75, 76].

### Eating disorder behaviors

Dietary restriction, purging, subjective binge eating, and objective binge eating were assessed by four related questions adapted from the Mini International Neuropsychiatric Interview—Anorexia Nervosa Criteria [55], which is used to screen ED in both clinical and epidemiological settings [16, 60, 61]. Previous studies [18, 60] use this inventory to measure diverse ED symptoms established according to DSM-IV axis I criteria (American Psychiatric Association, 1994). There is little evidence to suggest the changes between the DSM-IV and DSM-5 are significant, with research showing little change in a community sample (similar to the sample in this study) even with the introduction of Binge Eating Disorder, which is summed to the conservative changes between ED in the two versions [25]. Main changes from DSM-IV to DSM-5 result in a reduction of eating disorders not otherwise specified [6], a group excluded in this study, therefore the resource available (DSM-IV) was utilised. Dietary restriction was defined based on whether adolescents tried to avoid weight gain in the past three month (0 = no, 1 = yes). Purging was defined based on whether adolescents made themselves vomit or use laxatives to avoid weight increase after eating (0 = no, 1 = yes). Subjective binge eating and objective binge eating were measured by two items [7, 22]. Binary yes/no questions were asked about eating too much food in a short period of time and feeling out of control. Students were categorized as subjective binge eating if they ate too much food in a short period of time and felt under control. Students were categorized as having reported binge eating if they chose yes on either item.

### Suicidal ideation and non-suicidal self-harm

Suicidal ideation was measured by the item “have you had any suicidal ideation in the last month?”, with the option to choose from “no” or “yes”. Non-suicidal self-harm was measured by the item “I have tried to hurt myself deliberately without intention to kill myself in the last month”, choosing from “no” or “yes”. The two items have been used widely in previous studies [38, 39, 56].

### Reported cognitive deficits

The Reported Deficits Questionnaire (PDQ-5) was used to assess reported cognitive deficits, which can capture attention, memory, and executive function related-essential cognitive dysfunction [46]. PDQ-5 is a 5-item Likert scale and each item provides the option to choose from 0 (none) to 4 (very frequently). A higher mean score indicates more severe reported cognitive deficits. The Chinese versions of the PDQ-5 has good validity and reliability [19] and has been used previously with Chinese adolescents [9]. The Cronbach’s alpha was 0.78 in this survey.

### Anxiety symptoms

The Generalized Anxiety Disorder-7 (GAD-7) was used to screen for anxiety symptoms [62]. The GAD-7 is a 7-item, 4-point Likert scale, with the total scores ranging 0 from 21. A higher mean score indicates a higher level of anxiety symptoms and it has confirmed validity and reliability in Chinese populations [79], including adolescents [10, 78, 79] Cronbach’s alpha was 0.94 in this survey.

### Depression symptoms

The Patient Health Questionnaire-9 (PHQ-9) was used to screen for depression symptoms in this study [29]. The PHQ-9 consists of 9 items, and each item is scored from 0 (not at all) to 3 (nearly every day). A higher mean score indicates a higher level of depressive symptoms. The Chinese version of PHQ-9 has confirmed good validity and reliability [73]. Studies focused on adolescent populations also employ the Chinese version of PHQ-9 [78, 80]. The Cronbach’s alpha was 0.93 in this study.

### Reported health status

Reported health status was assessed by a single item adapted from the 36-Item Short -Form Survey (36-SF) [33]. The item asked, “In general, how what do you feel your health status is?”, and participants choose from excellent (scored 1) to poor (scored 5).

### School bullying experiences

Previous studies revealed that traumatic events in the school environment, especially school bullying experiences, were associated with ED [35, 36, 52, 67]. The school bullying experiences was measured by two questions: “Have you ever been bullied at school in this academic year?” and “Have you ever bullied or laughed at others in this academic year?”. Participants were coded as victims of school bullying if they answered “yes” to the first question. If the second question had a response of “yes”, the participants were coded as perpetrators of school bullying. The single item measurements of both school bullying victimization and perpetration have been widely used in large sample epidemiological surveys [23, 24].

### Statistical analysis

Basic socio-demographic variables, health related factors and status were depicted as Number (%) for categorical variables and Mean (*SD*) for continuous variables. All variables included in the current study (e.g., socio-demographic variables, sexual orientation, covariate variables, and ED behaviors) were compared between male and female adolescents using chi-square test with *φ* as the effect size for categorical variables, and t-test with Cohen’s *d* as the effect size for continuous variables. Binary logistic regressions were used to explore whether sexual orientation contributed to ED behaviors among students, with ED behaviors as the dependent variable, and socio-demographic characteristics and health related factors as the independent variables. Since heterosexuals are usually the majority group, we decided that the heterosexual orientation group would act as the reference group. Moreover, we conduct binary logistic regression to test the associations between the independent variables (excluding sexual orientation) and ED (results are presented in Additional file 1: Tables S1–S16 in the Appendix). The binary logistic regression models were run for both male and female adolescents separately. All analyses were conducted by Stata/SE 16.1 software and statical significance was set at the 0.05 (two-tailed) level in this study. We use the term “associations” in this study to mean the “correlate shown to precede the outcome”, instead of strictly indicating the causality. In addition, the male or female term used in sections of the analysis and results refers to one’s biological sex.

## Results

### Characteristics of the participants

The characteristics of the participants are shown in Table 1 and the mean age was 14.74 (*SD* = 1.46). Compared to female adolescents, male adolescents reported decreased anxiety symptoms (*t* = − 12.39, *p* < 0.001, Cohen’s *d* = − 0.233). Moreover, male adolescents were more likely to be the perpetrator in school bullying (χ^2^ = 190.61, *p* < 0.001, φ = 0.129). Additionally, male adolescents reported a lower likelihood of taking dietary restrictions (χ^2^ = 290.08, *p* < 0.001, φ = 0.160).

**Table 1:** Basic socio-demographic characteristics and health related features of samples

Variables	Total (N = 11,440)		Male adolescents (N = 6145)		Female adolescents (N = 5295)		χ^2^/*T*^b^	*P*	Effect size (Cohen's *d*/ *φ*)
	Mean ± *SD*	N (%)	Mean ± *SD*	N (%)	Mean ± *SD*	N (%)			
Age	14.74 ± 1.46	–	14.74 ± 1.45	–	14.75 ± 1.47	–	− 0.37	0.709	− 0.007
Household Registration Residence^a^							10.64	0.001	0.032
Rural	–	4207 (39.35)	–	2175 (37.92)	–	2032 (41.01)			
Urban	–	6484 (60.65)	–	3561 (62.08)	–	2923 (58.99)			
Being the Only Child^a^							76.81	< 0.001	0.083
No	–	7383 (65.89)	–	3742 (62.24)	–	3641 (70.11)			
Yes	–	3822 (34.11)	–	2270 (37.76)	–	1552 (29.89)			
Grade^a^							5.25	0.022	0.021
Junior middle school	–	8579 (75.03)	–	4662 (75.89)	–	3917 (74.03)			
Senior middle school	–	2855 (24.97)	–	1481 (24.11)	–	1374 (25.97)			
School residential status^a^							5.91	0.015	0.023
Board at school	–	2900 (25.74)	–	1502 (24.81)	–	1398 (26.82)			
Attend a day school	–	8365 (74.26)	–	4551 (75.19)	–	3814 (73.18)			
Sexual orientation							318.59	< 0.001	0.096
Heterosexual	–	4418 (38.62)	–	2767 (45.03)	–	1651 (31.18)			
Gay/Lesbian	–	653 (5.71)	–	241 (3.92)	–	412 (7.78)			
Bisexual	–	1524 (13.32)	–	628 (10.22)	–	896 (16.92)			
Reporting no sexual attraction	–	4845 (42.35)	–	2509 (40.83)	–	2336 (44.12)			
Suicidal Ideation							98.99	< 0.001	0.093
No	–	9749 (85.22)	–	5425 (88.28)	–	4324 (81.66)			
Yes	–	1691 (14.78)	–	720 (11.72)	–	971 (18.34)			
Non-suicidal self-Harm							85.68	< 0.001	0.087
No	–	10,061 (87.95)	–	5565 (90.56)	–	4496 (84.91)			
Yes	–	1379 (12.05)	–	580 (9.44)	–	799 (15.09)			
Perceived cognitive deficits^a^	1.23 ± 0.85	–	1.17 ± 0.86	–	1.30 ± 0.84	–	− 8.15	< 0.001	− 0.153
Anxiety symptoms^a^	0.74 ± 0.72	–	0.66 ± 0.70	–	0.82 ± 0.73	–	− 12.39	< 0.001	− 0.233
Depression symptoms^a^	0.59 ± 0.68	–	0.54 ± 0.65	–	0.65 ± 0.70	–	− 8.89	< 0.001	− 0.167
Poor self-rated health status^a^	2.09 ± 0.81	–	2.02 ± 0.83	–	2.18 ± 0.77	–	− 10.54	< 0.001	− 0.199
School bullying victim^*a*^									
No	–	10,549 (92.41)	–	5556 (90.68)	–	4993 (94.42)	56.65	< 0.001	0.070
Yes	–	866 (7.59)	–	571 (9.32)	–	295 (5.58)			
School bullying perpetrator^*a*^									
No	–	9583 (83.77)	–	4876 (79.35)	–	4707 (88.90)	190.61	< 0.001	0.129
Yes	–	1857 (16.23)	–	1269 (20.65)	–	588 (11.10)			
Dietary restriction^a^							290.08	< 0.001	0.160
No	–	6735 (59.03)	–	4063 (66.31)	–	2672 (50.59)			
Yes	–	4674 (40.97)	–	2064 (33.69)	–	2610 (49.41)			
Purging^a^							0.66	0.415	0.008
No	–	10,644 (93.69)	–	5719 (93.86)	–	4925 (93.49)			
Yes	–	717 (6.31)	–	374 (6.14)	–	343 (6.51)			
Subjective Binging^a^							42.51	< 0.001	0.061
No	–	10,122 (88.61)	–	5325 (86.81)	–	4797 (90.70)			
Yes	–	1301 (11.39)	–	809 (13.19)	–	492 (9.30)			
Binge Eating^a^							0.11	0.738	0.003
No	–	8629 (75.54)	–	4626 (75.42)	–	4003 (75.69)			
Yes	–	2794 (24.46)	–	1508 (24.58)	–	1286 (24.31)			

^a^Missing data were not entered statistical analysis

^b^χ^2^ tests or T tests were used to test the differences between male and female adolescents

### Associations between sexual orientation and ED behaviors

Tables 2, 3, 4, and 5 reveal the results of how sexual orientation is associated with ED behaviors for male and female adolescents separately. After controlling for the socio-demographic variables and associations, the binary logistic regression models revealed that, among male adolescents, being of an reporting no sexual attraction (heterosexual as ref.) was associated with lower likelihood of dietary restriction (*OR* 0.835; 95% CI 0.731–0.954; *p* < 0.01, Table 2), purging (*OR* 0.709; 95% CI 0.536–0.937; *p* < 0.05, Table 3), and binge eating (*OR* 0.852; 95% CI 0.731–0.993; *p* < 0.05, Table 5).

**Table 2:** Risk factors and association with dietary restriction in school adolescents

	Male adolescents					Female adolescents				
	Odds ratio	Std. err	*Z* value	*P* value	[95% conf. interval]	Odds ratio	Std. err	*Z* value	*P* value	[95% conf. interval]
*Sexual orientation (Heterosexual = 0)*										
Gay/Lesbian	0.827	0.132	− 1.195	0.232	0.605–1.129	1.136	0.139	1.045	0.296	
Bisexual	0.914	0.093	− 0.877	0.380	0.748–1.117	0.957	0.089	− 0.472	0.637	0.798–1.148
Reporting no sexual attraction sexuality	0.835**	0.057	− 2.658	0.008	0.731–0.954	0.695***	0.050	− 5.047	0.000	0.604–0.801
Age	0.982	0.022	− 0.823	0.411	0.940–1.026	1.077***	0.024	3.331	0.001	1.031–1.124
Household registration residence (Urban = 0)	0.983	0.060	− 0.280	0.779	0.872–1.108	0.946	0.059	− 0.889	0.374	0.837–1.069
Being the only child (No = 0)	1.011	0.061	0.176	0.860	0.897–1.139	0.944	0.063	− 0.866	0.387	0.828–1.076
Suicidal ideation (No = 0)	1.161	0.119	1.451	0.147	0.949–1.419	1.320**	0.127	2.892	0.004	1.094–1.593
Non-suicidal self-harm (No = 0)	1.105	0.121	0.910	0.363	0.891–1.371	1.566***	0.155	4.536	0.000	1.290–1.901
Perceived cognitive deficits	0.930	0.036	− 1.842	0.065	0.862–1.005	1.164***	0.049	3.579	0.000	1.071–1.265
Anxiety symptoms	1.056	0.078	0.735	0.462	0.914–1.219	1.139	0.083	1.801	0.072	0.989–1.313
Depression symptoms	1.175	0.097	1.951	0.051	0.999–1.382	1.007	0.084	0.089	0.929	0.856–1.186
Poor self-rated health status	1.405***	0.052	9.239	0.000	1.307–1.510	1.327***	0.056	6.746	0.000	1.222–1.440
School bullying victim (No = 0)	1.187	0.122	1.673	0.094	0.971–1.451	1.131	0.161	0.866	0.387	0.856–1.495
School bullying perpetrator (No = 0)	1.065	0.080	0.849	0.396	0.920–1.233	1.207	0.125	1.824	0.068	0.986–1.477
Constant	0.326***	0.110	− 3.326	0.001	0.169–0.631	0.137***	0.046	− 5.956	0.000	0.072–0.264
Observations			5441					4772		
DF			14					14		
Chi^2^			171.6					353.7		
Log likelihood			− 3386					− 3130		

***p < 0.001, **p < 0.01

**Table 3:** Risk factors and association with purging in school adolescents

	Male adolescents					Female adolescents				
	Odds ratio	Std. err	*Z* value	*P* value	[95% conf. interval]	Odds ratio	Std. err	*Z* value	*P* value	[95% conf. interval]
*Sexual orientation (Heterosexual = 0)*										
Gay/Lesbian	0.842	0.251	− 0.575	0.565	0.469–1.512	0.755	0.175	− 1.214	0.225	0.480–1.189
Bisexual	1.095	0.203	0.490	0.624	0.762–1.573	0.928	0.151	− 0.460	0.645	0.674–1.277
Reporting no sexual attraction	0.709*	0.101	− 2.417	0.016	0.536–0.937	0.557***	0.086	− 3.788	0.000	0.411–0.754
Age	0.975	0.042	− 0.574	0.566	0.896–1.062	1.103*	0.047	2.305	0.021	1.015–1.199
Household registration residence (Urban = 0)	1.161	0.141	1.225	0.221	0.914–1.473	1.156	0.145	1.151	0.250	0.903–1.479
Being the only child (No = 0)	0.923	0.115	− 0.642	0.521	0.724–1.178	1.066	0.142	0.476	0.634	0.820–1.384
Suicidal ideation (No = 0)	1.425*	0.246	2.051	0.040	1.016–2.000	1.246	0.202	1.359	0.174	0.907–1.711
Non-suicidal self-harm (No = 0)	1.368	0.246	1.748	0.080	0.963–1.945	1.513**	0.235	2.670	0.008	1.116–2.051
Perceived cognitive deficits	0.947	0.071	− 0.728	0.467	0.816–1.097	1.085	0.087	1.008	0.313	0.926–1.270
Anxiety symptoms	1.201	0.165	1.331	0.183	0.917–1.572	1.251	0.163	1.721	0.085	0.969–1.615
Depression symptoms	1.308	0.197	1.786	0.074	0.974–1.757	1.372*	0.199	2.188	0.029	1.034–1.823
Poor self-rated health status	1.287***	0.091	3.558	0.000	1.120–1.479	1.410***	0.123	3.926	0.000	1.188–1.674
School bullying victim (No = 0)	1.271	0.226	1.352	0.176	0.898–1.801	1.268	0.267	1.126	0.260	0.839–1.917
School bullying perpetrator (No = 0)	1.110	0.155	0.746	0.456	0.844–1.460	1.565**	0.254	2.763	0.006	1.139–2.150
Constant	0.037***	0.025	− 4.986	0.000	0.010–0.136	0.004***	0.002	− 8.590	0.000	0.001–0.013
Observations			5416					4758		
DF			14					14		
Chi^2^			117.1					232.4		
Log likelihood			− 1138					− 1019		

***p < 0.001, **p < 0.01, *p < 0.05

**Table 4:** Risk factors and association with subjective binging in school adolescents

	Male adolescents					Female adolescents				
	Odds ratio	Std. err	*Z* value	*P* value	[95% conf. interval]	Odds ratio	Std. err	*Z* value	*P* value	[95% conf. interval]
*Sexual orientation (Heterosexual = 0)*										
Gay/Lesbian	0.923	0.192	− 0.384	0.701	0.614–1.388	1.005	0.198	0.026	0.979	0.683–1.479
Bisexual	1.213	0.157	1.497	0.134	0.942–1.563	1.309	0.184	1.912	0.056	0.993–1.724
Reporting no sexual attraction	0.853	0.081	− 1.662	0.096	0.708–1.029	0.887	0.111	− 0.956	0.339	0.694–1.134
Age	1.019	0.030	0.617	0.537	0.961–1.080	1.097**	0.039	2.613	0.009	1.023–1.175
Household registration residence (Urban = 0)	1.040	0.087	0.476	0.634	0.884–1.225	0.990	0.103	− 0.092	0.927	0.807–1.215
Being the only child (No = 0)	0.952	0.080	− 0.583	0.560	0.808–1.122	1.028	0.114	0.252	0.801	0.827–1.278
Suicidal ideation (No = 0)	1.023	0.135	0.175	0.861	0.790–1.326	0.881	0.129	− 0.869	0.385	0.661–1.173
Non-suicidal self-harm (No = 0)	0.987	0.140	− 0.089	0.929	0.748–1.304	0.885	0.131	− 0.826	0.409	0.663–1.182
Perceived cognitive deficits	1.240***	0.062	4.279	0.000	1.124–1.369	1.278***	0.085	3.677	0.000	1.121–1.457
Anxiety symptoms	1.166	0.112	1.609	0.108	0.967–1.407	0.887	0.100	− 1.067	0.286	0.711–1.106
Depression symptoms	1.125	0.120	1.106	0.269	0.913–1.388	1.690***	0.210	4.216	0.000	1.324–2.158
Poor self-rated health status	1.067	0.053	1.318	0.187	0.969–1.176	1.097	0.078	1.316	0.188	0.956–1.260
School bullying victim (No = 0)	1.010	0.134	0.078	0.938	0.778–1.312	1.149	0.228	0.703	0.482	0.780–1.694
School bullying perpetrator (No = 0)	1.493***	0.140	4.286	0.000	1.243–1.794	1.370*	0.200	2.157	0.031	1.029–1.824
Constant	0.063***	0.029	− 6.089	0.000	0.026–0.153	0.011***	0.006	− 8.372	0.000	0.004–0.031
Observations			5443					4776		
DF			14					14		
Chi^2^			131.6					141.2		
Log likelihood			− 2100					− 1418		

***p < 0.001, **p < 0.01, *p < 0.05

**Table 5:** Risk factors and association with binge Eeting in school adolescents

	Male adolescents					Female adolescents				
	Odds ratio	Std. err	*Z* value	*P* value	[95% Conf. Interval]	Odds ratio	Std. Err	*Z* value	*P* value	[95% conf. interval]
*Sexual orientation (Heterosexual = 0)*										
Gay/Lesbian	1.083	0.183	0.470	0.638	0.777–1.509	1.377*	0.194	2.267	0.023	1.044–1.815
Bisexual	1.098	0.122	0.837	0.402	0.883–1.365	1.401**	0.150	3.153	0.002	1.136–1.727
Reporting no sexual attraction	0.852*	0.067	− 2.043	0.041	0.731–0.993	0.996	0.091	− 0.045	0.964	0.833–1.191
Age	0.983	0.025	− 0.683	0.495	0.936–1.033	1.062*	0.028	2.278	0.023	1.008–1.119
Household registration residence (Urban = 0)	1.042	0.072	0.602	0.547	0.910–1.194	0.991	0.076	− 0.118	0.906	0.853–1.151
Being the only child (No = 0)	0.892	0.062	− 1.644	0.100	0.778–1.022	1.039	0.085	0.471	0.637	0.886–1.219
Suicidal ideation (No = 0)	1.122	0.122	1.060	0.289	0.907–1.388	1.027	0.107	0.255	0.799	0.837–1.260
Non-suicidal self-harm (No = 0)	1.192	0.138	1.512	0.131	0.949–1.495	1.310**	0.137	2.579	0.010	1.067–1.609
Perceived cognitive deficits	1.405***	0.059	8.085	0.000	1.294–1.525	1.726***	0.086	11.013	0.000	1.566–1.902
Anxiety symptoms	1.108	0.088	1.291	0.197	0.948–1.294	1.057	0.087	0.671	0.502	0.899–1.242
Depression symptoms	1.445***	0.128	4.173	0.000	1.216–1.718	1.700***	0.157	5.728	0.000	1.418–2.038
Poor self-rated health status	1.220***	0.050	4.826	0.000	1.125–1.322	1.218***	0.062	3.856	0.000	1.102–1.347
School bullying victim (No = 0)	1.240*	0.135	1.973	0.048	1.001–1.534	1.315	0.199	1.811	0.070	0.978–1.769
School bullying perpetrator (No = 0)	1.601***	0.125	6.006	0.000	1.373–1.867	1.754***	0.192	5.129	0.000	1.415–2.174
Constant	0.120***	0.046	− 5.574	0.000	0.057–0.253	0.019***	0.008	− 9.779	0.000	0.009–0.042
Observations			5443					4776		
DF			14					14		
Chi^2^			502.3					765.6		
Log likelihood			− 2773					− 2269		

***p < 0.001, **p < 0.01, *p < 0.05

As for female adolescents, having an reporting no sexual attraction (heterosexual as reference) was significantly associated with being less likely to have dietary restriction (*OR* 0.695; 95% CI 0.604–0.801; *p* < 0.001, Table 2) and purging (*OR* 0.557; 95% CI 0.411–0.754; *p* < 0.001, Table 3). Bisexual female (heterosexual as reference) was positively correlated with objective binge eating (*OR* 1.401; 95% CI 1.136–1.727, *p* < 0.01, Table 5). Moreover, female adolescents who were lesbians (heterosexual as reference) had a higher likelihood of objective binge eating (*OR* 1.377; 95% CI 1.044–1.815, *p* < 0.05, Table 5).

### Associations between suicidal ideation and non-suicidal self-harm and ED behaviors

Among male adolescents, suicidal ideation was positively associated with purging (*OR* 1.425; 95% CI 1.016–2.000; *p* < 0.05, Table 3). As for female adolescents, suicidal ideation was associated with dietary restrictions (*OR* 1.320; 95% CI 1.094–1.593; *p* < 0.01, Table 2), non-suicidal self-harm was associated with dietary restriction (*OR* 1.566; 95% CI 1.290–1.901; *p* < 0.001, Table 2), purging (*OR* 1.513; 95% CI 1.116–2.051; *p* < 0.01, Table 3), and objective binge eating (*OR* 1.310; 95% CI 1.067–1.609; *p* < 0.01, Table 5).

### Associations between reported cognitive deficits and ED behaviors

Among male adolescents, reported cognitive deficits were associated with subjective binge eating (*OR* 1.240; 95% CI 1.124–1.369; *p* < 0.001, Table 4) and objective binge eating (*OR* 1.405; 95% CI 1.294–1.525; *p* < 0.001, Table 5). As for female adolescents, reported cognitive deficits were associated with dietary restriction (*OR* 1.164; 95% CI 1.071–1.265; *p* < 0.001, Table 2), subjective binge eating (*OR* 1.278; 95% CI 1.121–1.457; *p* < 0.001, Table 4), and objective binge eating (*OR* 1.726; 95% CI 1.566–1.902; *p* < 0.001, Table 5).

### Associations between anxiety and depressive symptoms and ED behaviors

Results revealed that, the severity of anxiety was not associated with ED behaviors for both male and female adolescents. Among male adolescents, severity of depression was associated with objective binge eating (*OR* 1.445; 95% CI 1.216–1.718; *p* < 0.001, Table 5). As for female adolescents, severity of depression was positively associated with the likelihood of purging (*OR* 1.372; 95% CI 1.034–1.823; *p* < 0.05, Table 3), subjective binge eating (*OR* 1.690; 95% CI 1.324–2.158;* p* < 0.001, Table 4), and objective binge eating (*OR* 1.700; 95% CI 1.418–2.038; *p* < 0.001, Table 5).

### Associations between reported health and ED behaviors

Among male adolescents, poorer self-rated health status was positively associated with dietary restriction (*OR* 1.405; 95% CI 1.307–1.510; *p* < 0.001, Table 2), purging (*OR* 1.287; 95% CI 1.120–1.479; *p* < 0.001, Table 3), and objective binge eating (*OR* 1.220; 95% CI 1.125–1.322; *p* < 0.001, Table 5). As for female adolescents, poor reported health status was associated with dietary restriction (*OR* 1.327; 95% CI 1.222–1.440; *p* < 0.001, Table 2), purging (*OR* 1.410; 95% CI 1.188–1.674; *p* < 0.001, Table 3), and objective binge eating (*OR* 1.218; 95% CI 1.102–1.347; *p* < 0.001, Table 5).

### Associations between school bullying and ED behaviors

Among male adolescents, being a perpetrator of school bullying was associated with subjective binge eating (*OR* 1.493; 95% CI 1.243–1.794; *p* < 0.001, Table 4) and objective binge eating (*OR* 1.601; 95% CI 1.373–1.867; *p* < 0.001, Table 5). Being a victim of school bullying was associated with objective binge eating (*OR* 1.240; 95% CI 1.001–1.534; *p* < 0.05, Table 5). As for female adolescents, being a perpetrator of school bullying was positively associated with the likelihood of purging (*OR* 1.565; 95% CI 1.139–2.150; *p* < 0.01, Table 3), subjective binge eating (*OR* 1.370; 95% CI 1.029–1.824; *p* < 0.05), and objective binge eating (*OR* 1.754; 95% CI 1.415–2.174; *p* < 0.001, Table 5).

## Discussion

This is the first comprehensive assessment of the associations across different ED behaviors in Chinese adolescents, which compared psychiatric symptoms and cognitive deficits between male and female adolescents in China. There is insufficient understanding on the underlying pathological mechanism of ED, particularly in sexual minority youth [26]. The lack of insight into associations for ED limits the intervention and prevention available [27, 59]. The results of this study begin to understand ED behaviors from a more comprehensive perspective, and it could provide useful information for clinical interventions and prevention methods. Our results revealed multiple associations with ED behaviors, which covered aspects of sexual orientation, suicidal ideation, self-harm, mental health symptoms, cognitive deficits, reported health status, and school bullying experiences.

### Associations between sexual orientation and ED

Significant differences in ED behaviors were found across different sexual orientations. Compared with the heterosexual orientation, being an reporting no sexual attraction was associated with less ED behaviors in both male and female adolescents in terms of both purging and dietary restriction. Female sexual minority groups demonstrated a higher risks of ED behaviors, with bisexual and lesbian female adolescents more likely to engage in objective binge eating. This is the first study to identify adolescents with an reporting no sexual attraction having less risk of engaging in ED behaviors, in both male and female adolescents. Noticeably, the female sexual minority groups demonstrated a higher risk of ED behaviors, with bisexual female adolescents more likely to engage in subjective binge eating and objective binge eating, and lesbian adolescents more likely to engage in objective binge eating. Unlike the comparison between general males and females, in which female adolescents showed significantly higher scores in dietary restriction, female sexual minority groups tended to behave in the opposite way by engaging in overeating and binge eating. This is inconsistent with previous finding that suggest when compared with heterosexual female adolescents, lesbian or bisexual female adolescents are just as likely as heterosexual female adolescents to feel pressure to conform to the ideal beauty standard [3, 66]. It is consistent though in confirming that lesbian adolescents are more likely to be obese due to overeating and binge eating than their heterosexual counterparts [66]. However, it is still important to be aware of the inconsistent research results in lesbian adolescents in relation to their body image and eating issues [71]. Such as, when other researchers indicate that lesbians are less invested in appearance and maintaining weight, and less concerned with dieting and thinness than heterosexual females or gay males [70], when the literature is still in its infancy.

### Associations between other factors and ED

Having depressive symptoms was pervasive in females across purging, subjective binge eating, and objective binge eating, with the only reported risk in males being objective binge eating. The results indicated that it is possible that female adolescents used ED behaviors as a maladaptive way of coping with depressive symptoms. Research has shown that depression is positively and directly associated with emotional eating, and depression is indirectly related to emotional eating via both alexithymia and impulsivity [50]. That is, there are also potential mediating pathways between depression and ED behaviors. The cultural ideal body image of thinness can cause depression among females [41], which could lead to continuation of ED behaviors. In China, slim female beauty has become a cultural preference and a fashionable female aesthetic standard [37]. This social pressure on this ideal female body could cause more distress for adolescent females, which could lead to an increase in ED behaviors. The study found that cognitive deficits were significantly associated with subjective binge eating and objective binge eating, this was true for both male and female adolescents. Although the role of cognitive deficits in the development of ED requires further research, researchers have proposed that cognitive deficits could pre-exist and underlie the aetiology of ED [32]. This study supports the evidence that it is necessary to investigate neuropsychological deficits as a potential association with ED onset, in relation to sex and sexual orientation.

A previous study found that objective binge eating was associated with greater impairment in males than females, and purging was associated with general health impairment for females, but higher general health for males [44]. Researchers proposed that males and females might have different perceptions about purging, and males may perceive the health benefits of purging to feel cleansed [44]. However, unlike the previous study, this study found that both objective binge eating and purging were associated with a poorer reported health status for both male and female adolescents. The shared protective factors in terms of purging were reporting no sexual attraction in male or female adolescents.

Previous study showed that there was an association between being the victims of bullying and binge-eating/purging [35]. Moreover, research also indicated that perpetrators of bullying also have adverse health outcomes [57]. Bullying is said to predict ED behaviors in both victims and perpetrators [12]. Our results were consistent with previous findings, while it also identified sex differences. Being a perpetrator of school bullying was significantly related with three ED behavior outcomes in female adolescents including purging, subjective binge eating, and binge eating. Being a school bullying perpetrator was also significantly related with subjective binge eating and binge eating in male adolescents. Across all the ED behaviors, binge eating was closely related with school bullying, with bullying perpetrators in both sexes showing a significant correlation with binge eating and being a bullying victim in male adolescents was significantly correlated with binge eating. The nature of the link between being bullied and ED behaviors is not clear [67]. Adolescent could get teased due to overweight body shaming, which could be caused by binge eating. It is also possible that adolescent use binge eating as way of coping with the stress caused by bullying.

### Limitations

There are several limitations that need to be considered when interpreting the results. First, due to the cross-sectional nature of the survey, no causal relationships of ED behaviors can be established. Future longitudinal studies are required to investigate the causes of ED behaviors in adolescents. Second, in the economic well-developed southern region of China, ED behaviors might be different from other regions in China that are more rural. The associations of ED behaviors identified in this study may therefore not represent other adolescents in economically deprived areas. Third, due to the limited resources, the current study did not measure all the associations with ED behaviors. For example, previous research suggests that low BMI was the most important predictor for onset of Anorexia Nervosa [63], and the researchers did not measure the BMI, which is known to be contested. Fourth, we did not include gender diverse adolescents in the current study as previous research suggests that transgender individuals may experience specific body related image dissatisfaction, which could be closely associated with EDs [47]. Moreover, issues related to being an intersex or transgender adolescent were not included in the current study, and is a point for future research. Fifth, the study investigated ED behaviors using scale measurement with a yes/no responses rather than a nuanced questionnaire or relying on a clinical diagnosis of ED. Thus, our results have limitations in capturing the granularity of ED behaviors. Future studies could adopt a more nuanced questionnaire design or focus on those diagnosed with ED, which could result in further impairments for adolescents. Sixth, given the sample analysed were quite young, the prevalence of sexual orientation ambiguity was high, which may lead to potential measurement error in identifying the group of reporting no sexual attraction adolescents. Thus, we cannot confirm the sexual orientation of these adolescents.

## Conclusion

Considering the unclear aetiology of ED and the high morbidity and mortality rates, the detection of ED at early stage is critical [17]. The research was conducted with a large school sample to identify associations between ED behaviors, and overeating, purging, and dietary restrictions. This is one of the first comprehensive studies to explore multiple ED behaviors in Chinese adolescents considering both sex and sexual orientation. In conclusion, this study revealed that the female sexual minority groups had the most associations with ED behaviors, and the reporting no sexual attraction adolescents tended to be less likely to be associated with ED behaviors. The findings could be used to provide rigorous evidence for future prevention measures and interventions in high-risk groups. We recommended clinicians implement tailored prevention strategies that are different for each of the adolescent groups studied, and that this needs to be specific to the different ED behaviors discussed.

## Supplementary Information

**Additional file 1**. Risk factors and association with eating disorder behaviors in school adolescents.

## Data Availability

The datasets used and/or analysed during the current study are available from the corresponding author on reasonable request.
