# Supplementary material for: Associations between sex differences, eating disorder behaviors, physical and mental health, and self-harm among Chinese adolescents
Source: J Eat Disord. 2023 Feb 27;11:30. doi: 10.1186/s40337-023-00754-7 (PMC9972801; doi:10.1186/s40337-023-00754-7)
Supplement: Supplementary file 1 — Additional file 1. Risk factors and association with eating disorder behaviors in school adolescents. [file 40337_2023_754_MOESM1_ESM.docx]

**Table S1: Risk Factors and Association with Dietary Restriction in Heterosexual School Adolescents**

|  | Male adolescents | | | | | Female adolescents | | | | |
| --- | --- | --- | --- | --- | --- | --- | --- | --- | --- | --- |
|  | Odds Ratio | Std. Err. | *Z* value | *P* value | [95% Conf. Interval] | Odds Ratio | Std. Err. | *Z* value | *P* value | [95% Conf. Interval] |
| Age | 0.967 | 0.028 | -1.151 | 0.250 | 0.914 - 1.024 | 1.137*** | 0.043 | 3.400 | 0.001 | 1.056 - 1.224 |
| Household Registration Residence (Urban=0) | 0.971 | 0.086 | -0.327 | 0.744 | 0.816 - 1.156 | 1.051 | 0.117 | 0.444 | 0.657 | 0.844 - 1.307 |
| Being the Only Child (No=0) | 1.144 | 0.100 | 1.537 | 0.124 | 0.964 - 1.358 | 0.827 | 0.095 | -1.650 | 0.099 | 0.660 - 1.036 |
| Suicidal Ideation (No=0) | 1.241 | 0.184 | 1.454 | 0.146 | 0.928 - 1.660 | 1.349 | 0.236 | 1.707 | 0.088 | 0.957 - 1.902 |
| Non-Suicidal Self-Harm (No=0) | 1.148 | 0.180 | 0.880 | 0.379 | 0.844 - 1.560 | 1.526* | 0.277 | 2.326 | 0.020 | 1.069 - 2.178 |
| Perceived Cognitive Deficits | 0.920 | 0.054 | -1.416 | 0.157 | 0.820 - 1.032 | 1.153 | 0.091 | 1.799 | 0.072 | 0.987 - 1.347 |
| Anxiety Symptoms | 1.187 | 0.124 | 1.638 | 0.101 | 0.967 - 1.457 | 1.308* | 0.178 | 1.976 | 0.048 | 1.002 - 1.708 |
| Depression Symptoms | 0.991 | 0.118 | -0.075 | 0.940 | 0.785 - 1.251 | 0.914 | 0.144 | -0.573 | 0.567 | 0.671 - 1.245 |
| Poor Self-rated Health Status | 1.430*** | 0.079 | 6.487 | 0.000 | 1.284 - 1.594 | 1.383*** | 0.107 | 4.214 | 0.000 | 1.189 - 1.609 |
| School Bullying Victim (No=0) | 1.118 | 0.173 | 0.718 | 0.473 | 0.825 - 1.514 | 1.505 | 0.417 | 1.473 | 0.141 | 0.874 - 2.591 |
| School Bullying Perpetrator (No=0) | 1.005 | 0.104 | 0.047 | 0.963 | 0.820 - 1.231 | 1.551* | 0.293 | 2.325 | 0.020 | 1.071 - 2.246 |
| Constant | 0.392* | 0.171 | -2.144 | 0.032 | 0.166 - 0.923 | 0.051*** | 0.028 | -5.319 | 0.000 | 0.017 - 0.152 |
| Observations | 2,474 | | | | | 1,503 | | | | |
| DF | 11 | | | | | 11 | | | | |
| Chi^2^ | 70.39 | | | | | 119.8 | | | | |
| Log likelihood | -1576 | | | | | -977.2 | | | | |

*** p<0.001, ** p<0.01, * p<0.05**Table S2: Risk Factors and Association with Purging in Heterosexual School Adolescents**

|  | Male adolescents | | | | | Female adolescents | | | | |
| --- | --- | --- | --- | --- | --- | --- | --- | --- | --- | --- |
|  | Odds Ratio | Std. Err. | *Z* value | *P* value | [95% Conf. Interval] | Odds Ratio | Std. Err. | *Z* value | *P* value | [95% Conf. Interval] |
| Age | 0.961 | 0.054 | -0.711 | 0.477 | 0.861 - 1.072 | 1.132 | 0.078 | 1.808 | 0.071 | 0.990 - 1.296 |
| Household Registration Residence (Urban=0) | 1.269 | 0.216 | 1.400 | 0.161 | 0.909 - 1.771 | 1.048 | 0.214 | 0.229 | 0.819 | 0.703 - 1.562 |
| Being the Only Child (No=0) | 1.017 | 0.173 | 0.098 | 0.922 | 0.728 - 1.421 | 0.889 | 0.193 | -0.541 | 0.589 | 0.581 - 1.361 |
| Suicidal Ideation (No=0) | 1.395 | 0.344 | 1.349 | 0.177 | 0.860 - 2.261 | 1.648* | 0.419 | 1.964 | 0.050 | 1.001 - 2.713 |
| Non-Suicidal Self-Harm (No=0) | 1.467 | 0.367 | 1.534 | 0.125 | 0.899 - 2.396 | 1.427 | 0.361 | 1.408 | 0.159 | 0.870 - 2.341 |
| Perceived Cognitive Deficits | 0.837 | 0.094 | -1.580 | 0.114 | 0.671 - 1.044 | 0.998 | 0.136 | -0.013 | 0.989 | 0.765 - 1.303 |
| Anxiety Symptoms | 1.335 | 0.254 | 1.520 | 0.128 | 0.920 - 1.938 | 1.418 | 0.311 | 1.594 | 0.111 | 0.923 - 2.180 |
| Depression Symptoms | 1.289 | 0.272 | 1.204 | 0.228 | 0.853 - 1.948 | 1.200 | 0.297 | 0.738 | 0.461 | 0.739 - 1.951 |
| Poor Self-rated Health Status | 1.307** | 0.135 | 2.591 | 0.010 | 1.067 - 1.600 | 1.570** | 0.229 | 3.097 | 0.002 | 1.180 - 2.089 |
| School Bullying Victim (No=0) | 1.413 | 0.364 | 1.342 | 0.180 | 0.853 - 2.341 | 1.508 | 0.526 | 1.177 | 0.239 | 0.761 - 2.988 |
| School Bullying Perpetrator (No=0) | 0.834 | 0.168 | -0.902 | 0.367 | 0.563 - 1.237 | 1.813* | 0.469 | 2.300 | 0.021 | 1.092 - 3.010 |
| Constant | 0.048*** | 0.041 | -3.581 | 0.000 | 0.009 - 0.253 | 0.002*** | 0.002 | -5.907 | 0.000 | 0.000 - 0.016 |
| Observations | 2,457 | | | | | 1,495 | | | | |
| DF | 11 | | | | | 11 | | | | |
| Chi^2^ | 53.37 | | | | | 86.92 | | | | |
| Log likelihood | -565 | | | | | -376.7 | | | | |

*** p<0.001, ** p<0.01, * p<0.05

**Table S3: Risk Factors and Association with Subjective Binging in Heterosexual School Adolescents**

|  | Male adolescents | | | | | Female adolescents | | | | |
| --- | --- | --- | --- | --- | --- | --- | --- | --- | --- | --- |
|  | Odds Ratio | Std. Err. | *Z* value | *P* value | [95% Conf. Interval] | Odds Ratio | Std. Err. | *Z* value | *P* value | [95% Conf. Interval] |
| Age | 0.938 | 0.037 | -1.635 | 0.102 | 0.868 - 1.013 | 1.023 | 0.063 | 0.364 | 0.716 | 0.906 - 1.155 |
| Household Registration Residence (Urban=0) | 1.225 | 0.145 | 1.716 | 0.086 | 0.972 - 1.546 | 0.921 | 0.170 | -0.443 | 0.658 | 0.641 - 1.324 |
| Being the Only Child (No=0) | 0.981 | 0.117 | -0.158 | 0.874 | 0.777 - 1.239 | 1.131 | 0.214 | 0.650 | 0.516 | 0.780 - 1.638 |
| Suicidal Ideation (No=0) | 0.704 | 0.141 | -1.759 | 0.079 | 0.476 - 1.041 | 0.898 | 0.228 | -0.423 | 0.672 | 0.546 - 1.478 |
| Non-Suicidal Self-Harm (No=0) | 0.935 | 0.191 | -0.329 | 0.742 | 0.626 - 1.396 | 1.090 | 0.278 | 0.338 | 0.736 | 0.661 - 1.798 |
| Perceived Cognitive Deficits | 1.246** | 0.094 | 2.921 | 0.003 | 1.075 - 1.444 | 1.437** | 0.174 | 2.992 | 0.003 | 1.133 - 1.823 |
| Anxiety Symptoms | 0.970 | 0.132 | -0.221 | 0.825 | 0.743 - 1.268 | 1.047 | 0.215 | 0.225 | 0.822 | 0.701 - 1.565 |
| Depression Symptoms | 1.613** | 0.243 | 3.177 | 0.001 | 1.201 - 2.167 | 1.384 | 0.322 | 1.400 | 0.162 | 0.878 - 2.182 |
| Poor Self-rated Health Status | 1.134 | 0.083 | 1.726 | 0.084 | 0.983 - 1.308 | 1.127 | 0.144 | 0.940 | 0.347 | 0.878 - 1.447 |
| School Bullying Victim (No=0) | 0.856 | 0.175 | -0.764 | 0.445 | 0.574 - 1.276 | 1.724 | 0.569 | 1.652 | 0.098 | 0.903 - 3.291 |
| School Bullying Perpetrator (No=0) | 1.425** | 0.186 | 2.720 | 0.007 | 1.104 - 1.840 | 1.153 | 0.302 | 0.544 | 0.587 | 0.690 - 1.925 |
| Constant | 0.177** | 0.105 | -2.915 | 0.004 | 0.055 - 0.567 | 0.024*** | 0.022 | -4.050 | 0.000 | 0.004 - 0.145 |
| Observations | 2,474 | | | | | 1,499 | | | | |
| DF | 11 | | | | | 11 | | | | |
| Chi^2^ | 69.27 | | | | | 45.21 | | | | |
| Log likelihood | -998.8 | | | | | -453.8 | | | | |

*** p<0.001, ** p<0.01, * p<0.05

**Table S4: Risk Factors and Association with Binge Eating in Heterosexual School Adolescents**

|  | Male adolescents | | | | | Female adolescents | | | | |
| --- | --- | --- | --- | --- | --- | --- | --- | --- | --- | --- |
|  | Odds Ratio | Std. Err. | *Z* value | *P* value | [95% Conf. Interval] | Odds Ratio | Std. Err. | *Z* value | *P* value | [95% Conf. Interval] |
| Age | 0.920* | 0.030 | -2.521 | 0.012 | 0.863 - 0.982 | 1.031 | 0.048 | 0.668 | 0.504 | 0.942 - 1.130 |
| Household Registration Residence (Urban=0) | 1.147 | 0.114 | 1.379 | 0.168 | 0.944 - 1.395 | 1.037 | 0.143 | 0.264 | 0.791 | 0.792 - 1.358 |
| Being the Only Child (No=0) | 0.906 | 0.090 | -0.989 | 0.323 | 0.745 - 1.102 | 1.085 | 0.155 | 0.571 | 0.568 | 0.820 - 1.437 |
| Suicidal Ideation (No=0) | 0.938 | 0.150 | -0.402 | 0.688 | 0.685 - 1.283 | 1.031 | 0.194 | 0.160 | 0.873 | 0.713 - 1.490 |
| Non-Suicidal Self-Harm (No=0) | 1.095 | 0.183 | 0.542 | 0.588 | 0.789 - 1.519 | 1.593* | 0.300 | 2.469 | 0.014 | 1.101 - 2.306 |
| Perceived Cognitive Deficits | 1.337*** | 0.085 | 4.560 | 0.000 | 1.180 - 1.514 | 1.894*** | 0.177 | 6.847 | 0.000 | 1.578 - 2.274 |
| Anxiety Symptoms | 0.982 | 0.112 | -0.161 | 0.872 | 0.785 - 1.228 | 1.260 | 0.195 | 1.495 | 0.135 | 0.931 - 1.706 |
| Depression Symptoms | 1.869*** | 0.239 | 4.898 | 0.000 | 1.455 - 2.401 | 1.301 | 0.230 | 1.488 | 0.137 | 0.920 - 1.840 |
| Poor Self-rated Health Status | 1.364*** | 0.084 | 5.049 | 0.000 | 1.209 - 1.538 | 1.273* | 0.121 | 2.546 | 0.011 | 1.057 - 1.534 |
| School Bullying Victim (No=0) | 1.291 | 0.210 | 1.574 | 0.115 | 0.939 - 1.775 | 1.406 | 0.393 | 1.219 | 0.223 | 0.813 - 2.433 |
| School Bullying Perpetrator (No=0) | 1.511*** | 0.166 | 3.766 | 0.000 | 1.219 - 1.874 | 2.224*** | 0.427 | 4.161 | 0.000 | 1.526 - 3.240 |
| Constant | 0.258** | 0.128 | -2.731 | 0.006 | 0.097 - 0.682 | 0.022*** | 0.015 | -5.494 | 0.000 | 0.006 - 0.085 |
| Observations | 2,474 | | | | | 1,499 | | | | |
| DF | 11 | | | | | 11 | | | | |
| Chi^2^ | 239.6 | | | | | 229.3 | | | | |
| Log likelihood | -1304 | | | | | -701.2 | | | | |

*** p<0.001, ** p<0.01, * p<0.05**Table S5: Risk Factors and Association with Dietary Restriction in Gay/Lesbian School Adolescents**

|  | Male adolescents | | | | | Female adolescents | | | | |
| --- | --- | --- | --- | --- | --- | --- | --- | --- | --- | --- |
|  | Odds Ratio | Std. Err. | *Z* value | *P* value | [95% Conf. Interval] | Odds Ratio | Std. Err. | *Z* value | *P* value | [95% Conf. Interval] |
| Age | 1.099 | 0.119 | 0.872 | 0.383 | 0.889 - 1.358 | 0.965 | 0.076 | -0.447 | 0.655 | 0.827 - 1.126 |
| Household Registration Residence (Urban=0) | 1.462 | 0.462 | 1.201 | 0.230 | 0.787 - 2.715 | 0.997 | 0.223 | -0.016 | 0.988 | 0.642 - 1.546 |
| Being the Only Child (No=0) | 1.364 | 0.466 | 0.909 | 0.363 | 0.699 - 2.663 | 1.117 | 0.274 | 0.451 | 0.652 | 0.691 - 1.806 |
| Suicidal Ideation (No=0) | 0.907 | 0.409 | -0.217 | 0.829 | 0.374 - 2.196 | 1.286 | 0.422 | 0.766 | 0.444 | 0.676 - 2.445 |
| Non-Suicidal Self-Harm (No=0) | 1.623 | 0.794 | 0.990 | 0.322 | 0.622 - 4.232 | 1.260 | 0.434 | 0.671 | 0.502 | 0.642 - 2.473 |
| Perceived Cognitive Deficits | 0.974 | 0.175 | -0.146 | 0.884 | 0.685 - 1.385 | 1.131 | 0.166 | 0.839 | 0.401 | 0.848 - 1.509 |
| Anxiety Symptoms | 1.443 | 0.469 | 1.129 | 0.259 | 0.763 - 2.729 | 1.480 | 0.367 | 1.580 | 0.114 | 0.910 - 2.407 |
| Depression Symptoms | 0.876 | 0.342 | -0.339 | 0.735 | 0.408 - 1.881 | 0.786 | 0.225 | -0.838 | 0.402 | 0.448 - 1.379 |
| Poor Self-rated Health Status | 1.357 | 0.235 | 1.764 | 0.078 | 0.967 - 1.904 | 1.225 | 0.184 | 1.347 | 0.178 | 0.912 - 1.645 |
| School Bullying Victim (No=0) | 1.862 | 0.878 | 1.319 | 0.187 | 0.739 - 4.691 | 1.901 | 0.984 | 1.240 | 0.215 | 0.689 - 5.244 |
| School Bullying Perpetrator (No=0) | 0.765 | 0.296 | -0.693 | 0.488 | 0.358 - 1.633 | 0.946 | 0.298 | -0.175 | 0.861 | 0.511 - 1.753 |
| Constant | 0.037* | 0.058 | -2.091 | 0.037 | 0.002 - 0.813 | 0.896 | 1.006 | -0.098 | 0.922 | 0.099 - 8.093 |
| Observations | 204 | | | | | 370 | | | | |
| DF | 11 | | | | | 11 | | | | |
| Chi^2^ | 14.55 | | | | | 16.42 | | | | |
| Log likelihood | -122.6 | | | | | -243.7 | | | | |

*** p<0.001, ** p<0.01, * p<0.05**Table S6: Risk Factors and Association with Purging in Gay/Lesbian School Adolescents**

|  | Male adolescents | | | | | Female adolescents | | | | |
| --- | --- | --- | --- | --- | --- | --- | --- | --- | --- | --- |
|  | Odds Ratio | Std. Err. | *Z* value | *P* value | [95% Conf. Interval] | Odds Ratio | Std. Err. | *Z* value | *P* value | [95% Conf. Interval] |
| Age | 1.028 | 0.200 | 0.143 | 0.886 | 0.702 - 1.505 | 0.857 | 0.146 | -0.907 | 0.364 | 0.613 - 1.197 |
| Household Registration Residence (Urban=0) | 0.681 | 0.438 | -0.598 | 0.550 | 0.193 - 2.405 | 1.252 | 0.549 | 0.512 | 0.609 | 0.530 - 2.956 |
| Being the Only Child (No=0) | 3.970* | 2.694 | 2.032 | 0.042 | 1.050 - 15.007 | 0.532 | 0.293 | -1.145 | 0.252 | 0.181 - 1.567 |
| Suicidal Ideation (No=0) | 3.169 | 2.433 | 1.503 | 0.133 | 0.704 - 14.269 | 0.727 | 0.468 | -0.494 | 0.621 | 0.206 - 2.570 |
| Non-Suicidal Self-Harm (No=0) | 0.925 | 0.796 | -0.090 | 0.928 | 0.171 - 4.995 | 3.826* | 2.228 | 2.304 | 0.021 | 1.222 - 11.980 |
| Perceived Cognitive Deficits | 0.890 | 0.305 | -0.341 | 0.733 | 0.454 - 1.743 | 1.451 | 0.390 | 1.385 | 0.166 | 0.857 - 2.458 |
| Anxiety Symptoms | 1.923 | 1.100 | 1.143 | 0.253 | 0.627 - 5.898 | 0.987 | 0.413 | -0.031 | 0.976 | 0.435 - 2.242 |
| Depression Symptoms | 0.668 | 0.435 | -0.620 | 0.535 | 0.186 - 2.394 | 1.099 | 0.557 | 0.186 | 0.853 | 0.407 - 2.966 |
| Poor Self-rated Health Status | 1.096 | 0.339 | 0.296 | 0.767 | 0.598 - 2.008 | 1.213 | 0.352 | 0.667 | 0.505 | 0.687 - 2.142 |
| School Bullying Victim (No=0) | 1.308 | 1.054 | 0.334 | 0.739 | 0.270 - 6.340 | 0.294 | 0.256 | -1.404 | 0.160 | 0.053 - 1.624 |
| School Bullying Perpetrator (No=0) | 2.075 | 1.375 | 1.102 | 0.271 | 0.566 - 7.606 | 5.167*** | 2.354 | 3.606 | 0.000 | 2.116 - 12.617 |
| Constant | 0.010 | 0.028 | -1.626 | 0.104 | 0.000 - 2.578 | 0.130 | 0.314 | -0.846 | 0.398 | 0.001 - 14.632 |
| Observations | 204 | | | | | 369 | | | | |
| DF | 11 | | | | | 11 | | | | |
| Chi^2^ | 17.93 | | | | | 30.84 | | | | |
| Log likelihood | -42.05 | | | | | -81.17 | | | | |

*** p<0.001, ** p<0.01, * p<0.05

**Table S7: Risk Factors and Association with Subjective Binging in Gay/Lesbian School Adolescents**

|  | Male adolescents | | | | | Female adolescents | | | | |
| --- | --- | --- | --- | --- | --- | --- | --- | --- | --- | --- |
|  | Odds Ratio | Std. Err. | *Z* value | *P* value | [95% Conf. Interval] | Odds Ratio | Std. Err. | *Z* value | *P* value | [95% Conf. Interval] |
| Age | 0.924 | 0.136 | -0.535 | 0.593 | 0.693 - 1.234 | 1.236 | 0.152 | 1.724 | 0.085 | 0.971 - 1.573 |
| Household Registration Residence (Urban=0) | 1.232 | 0.519 | 0.496 | 0.620 | 0.540 - 2.815 | 0.967 | 0.357 | -0.092 | 0.927 | 0.468 - 1.995 |
| Being the Only Child (No=0) | 1.423 | 0.647 | 0.776 | 0.438 | 0.584 - 3.470 | 0.931 | 0.381 | -0.174 | 0.862 | 0.418 - 2.075 |
| Suicidal Ideation (No=0) | 1.463 | 0.827 | 0.673 | 0.501 | 0.483 - 4.427 | 0.524 | 0.280 | -1.211 | 0.226 | 0.184 - 1.491 |
| Non-Suicidal Self-Harm (No=0) | 0.281 | 0.214 | -1.669 | 0.095 | 0.063 - 1.247 | 2.341 | 1.150 | 1.730 | 0.084 | 0.893 - 6.133 |
| Perceived Cognitive Deficits | 1.859** | 0.409 | 2.816 | 0.005 | 1.207 - 2.862 | 0.676 | 0.166 | -1.597 | 0.110 | 0.418 - 1.093 |
| Anxiety Symptoms | 0.782 | 0.335 | -0.574 | 0.566 | 0.337 - 1.813 | 1.271 | 0.467 | 0.652 | 0.515 | 0.618 - 2.612 |
| Depression Symptoms | 0.977 | 0.476 | -0.047 | 0.963 | 0.376 - 2.539 | 1.887 | 0.821 | 1.459 | 0.144 | 0.804 - 4.427 |
| Poor Self-rated Health Status | 1.038 | 0.227 | 0.173 | 0.863 | 0.677 - 1.593 | 1.312 | 0.336 | 1.062 | 0.288 | 0.795 - 2.167 |
| School Bullying Victim (No=0) | 1.992 | 1.152 | 1.190 | 0.234 | 0.641 - 6.191 | 0.422 | 0.346 | -1.053 | 0.292 | 0.085 - 2.104 |
| School Bullying Perpetrator (No=0) | 1.410 | 0.689 | 0.703 | 0.482 | 0.541 - 3.676 | 1.070 | 0.524 | 0.138 | 0.890 | 0.410 - 2.794 |
| Constant | 0.160 | 0.337 | -0.871 | 0.384 | 0.003 - 9.892 | 0.002*** | 0.004 | -3.417 | 0.001 | 0.000 - 0.070 |
| Observations | 205 | | | | | 371 | | | | |
| DF | 11 | | | | | 11 | | | | |
| Chi^2^ | 15.45 | | | | | 22.56 | | | | |
| Log likelihood | -79.36 | | | | | -111.3 | | | | |

*** p<0.001, ** p<0.01, * p<0.05

**Table S8: Risk Factors and Association with Binge Eating in Gay/Lesbian School Adolescents**

|  | Male adolescents | | | | | Female adolescents | | | | |
| --- | --- | --- | --- | --- | --- | --- | --- | --- | --- | --- |
|  | Odds Ratio | Std. Err. | *Z* value | *P* value | [95% Conf. Interval] | Odds Ratio | Std. Err. | *Z* value | *P* value | [95% Conf. Interval] |
| Age | 1.102 | 0.126 | 0.846 | 0.397 | 0.880 - 1.380 | 1.075 | 0.094 | 0.828 | 0.408 | 0.906 - 1.275 |
| Household Registration Residence (Urban=0) | 1.656 | 0.574 | 1.454 | 0.146 | 0.839 - 3.267 | 0.847 | 0.217 | -0.650 | 0.516 | 0.513 - 1.398 |
| Being the Only Child (No=0) | 1.481 | 0.543 | 1.070 | 0.285 | 0.721 - 3.040 | 0.993 | 0.275 | -0.026 | 0.979 | 0.577 - 1.709 |
| Suicidal Ideation (No=0) | 2.390 | 1.102 | 1.889 | 0.059 | 0.968 - 5.899 | 0.808 | 0.280 | -0.615 | 0.538 | 0.409 - 1.594 |
| Non-Suicidal Self-Harm (No=0) | 0.847 | 0.434 | -0.323 | 0.747 | 0.310 - 2.313 | 1.906 | 0.670 | 1.835 | 0.066 | 0.957 - 3.797 |
| Perceived Cognitive Deficits | 2.168*** | 0.414 | 4.050 | 0.000 | 1.491 - 3.152 | 1.502* | 0.240 | 2.546 | 0.011 | 1.098 - 2.054 |
| Anxiety Symptoms | 0.889 | 0.312 | -0.336 | 0.737 | 0.447 - 1.767 | 0.951 | 0.251 | -0.190 | 0.850 | 0.566 - 1.597 |
| Depression Symptoms | 1.018 | 0.414 | 0.043 | 0.966 | 0.459 - 2.257 | 2.259** | 0.687 | 2.678 | 0.007 | 1.244 - 4.101 |
| Poor Self-rated Health Status | 1.133 | 0.208 | 0.680 | 0.497 | 0.791 - 1.624 | 1.167 | 0.198 | 0.909 | 0.363 | 0.836 - 1.629 |
| School Bullying Victim (No=0) | 1.388 | 0.695 | 0.654 | 0.513 | 0.520 - 3.705 | 0.661 | 0.355 | -0.771 | 0.441 | 0.231 - 1.894 |
| School Bullying Perpetrator (No=0) | 1.268 | 0.511 | 0.591 | 0.555 | 0.576 - 2.792 | 1.132 | 0.383 | 0.367 | 0.713 | 0.583 - 2.199 |
| Constant | 0.014* | 0.024 | -2.526 | 0.012 | 0.001 - 0.384 | 0.031** | 0.040 | -2.737 | 0.006 | 0.003 - 0.374 |
| Observations | 205 | | | | | 371 | | | | |
| DF | 11 | | | | | 11 | | | | |
| Chi^2^ | 42.03 | | | | | 66.36 | | | | |
| Log likelihood | -107.8 | | | | | -198.8 | | | | |

*** p<0.001, ** p<0.01, * p<0.05**Table S9: Risk Factors and Association with Dietary Restriction in Bisexual School Adolescents**

|  | Male adolescents | | | | | Female adolescents | | | | |
| --- | --- | --- | --- | --- | --- | --- | --- | --- | --- | --- |
|  | Odds Ratio | Std. Err. | *Z* value | *P* value | [95% Conf. Interval] | Odds Ratio | Std. Err. | *Z* value | *P* value | [95% Conf. Interval] |
| Age | 1.176 | 0.099 | 1.919 | 0.055 | 0.997 - 1.388 | 1.122* | 0.061 | 2.121 | 0.034 | 1.009 - 1.248 |
| Household Registration Residence (Urban=0) | 0.611 | 0.154 | -1.948 | 0.051 | 0.372 - 1.003 | 0.661** | 0.104 | -2.635 | 0.008 | 0.486 - 0.899 |
| Being the Only Child (No=0) | 0.760 | 0.186 | -1.119 | 0.263 | 0.470 - 1.229 | 1.128 | 0.189 | 0.717 | 0.473 | 0.812 - 1.566 |
| Suicidal Ideation (No=0) | 1.940* | 0.595 | 2.160 | 0.031 | 1.063 - 3.539 | 1.198 | 0.252 | 0.858 | 0.391 | 0.793 - 1.810 |
| Non-Suicidal Self-Harm (No=0) | 1.349 | 0.475 | 0.851 | 0.395 | 0.677 - 2.691 | 1.319 | 0.278 | 1.312 | 0.189 | 0.872 - 1.993 |
| Perceived Cognitive Deficits | 1.032 | 0.145 | 0.225 | 0.822 | 0.783 - 1.360 | 1.299** | 0.131 | 2.590 | 0.010 | 1.066 - 1.584 |
| Anxiety Symptoms | 1.781* | 0.514 | 1.999 | 0.046 | 1.011 - 3.135 | 0.797 | 0.134 | -1.351 | 0.177 | 0.573 - 1.108 |
| Depression Symptoms | 0.607 | 0.198 | -1.530 | 0.126 | 0.321 - 1.151 | 1.533* | 0.280 | 2.342 | 0.019 | 1.072 - 2.192 |
| Poor Self-rated Health Status | 1.070 | 0.154 | 0.466 | 0.641 | 0.806 - 1.420 | 1.277* | 0.138 | 2.260 | 0.024 | 1.033 - 1.579 |
| School Bullying Victim (No=0) | 0.806 | 0.274 | -0.634 | 0.526 | 0.414 - 1.571 | 0.616 | 0.198 | -1.504 | 0.133 | 0.328 - 1.158 |
| School Bullying Perpetrator (No=0) | 1.160 | 0.301 | 0.571 | 0.568 | 0.697 - 1.930 | 1.214 | 0.258 | 0.913 | 0.361 | 0.801 - 1.841 |
| Constant | 0.016*** | 0.019 | -3.447 | 0.001 | 0.001 - 0.166 | 0.081** | 0.063 | -3.231 | 0.001 | 0.018 - 0.372 |
| Observations | 549 | | | | | 798 | | | | |
| DF | 11 | | | | | 11 | | | | |
| Chi^2^ | 21.66 | | | | | 70.67 | | | | |
| Log likelihood | -246.7 | | | | | -512 | | | | |

*** p<0.001, ** p<0.01, * p<0.05**Table S10: Risk Factors and Association with Purging in Bisexual School Adolescents**

|  | Male adolescents | | | | | Female adolescents | | | | |
| --- | --- | --- | --- | --- | --- | --- | --- | --- | --- | --- |
|  | Odds Ratio | Std. Err. | *Z* value | *P* value | [95% Conf. Interval] | Odds Ratio | Std. Err. | *Z* value | *P* value | [95% Conf. Interval] |
| Age | 0.997 | 0.120 | -0.021 | 0.983 | 0.788 - 1.263 | 1.164 | 0.097 | 1.817 | 0.069 | 0.988 - 1.372 |
| Household Registration Residence (Urban=0) | 0.942 | 0.325 | -0.173 | 0.862 | 0.479 - 1.852 | 1.104 | 0.295 | 0.372 | 0.710 | 0.655 - 1.863 |
| Being the Only Child (No=0) | 0.841 | 0.296 | -0.493 | 0.622 | 0.422 - 1.676 | 1.555 | 0.415 | 1.654 | 0.098 | 0.922 - 2.623 |
| Suicidal Ideation (No=0) | 2.077 | 0.840 | 1.808 | 0.071 | 0.940 - 4.588 | 1.006 | 0.341 | 0.017 | 0.987 | 0.518 - 1.953 |
| Non-Suicidal Self-Harm (No=0) | 1.225 | 0.550 | 0.451 | 0.652 | 0.507 - 2.955 | 0.943 | 0.300 | -0.185 | 0.853 | 0.505 - 1.760 |
| Perceived Cognitive Deficits | 1.117 | 0.211 | 0.585 | 0.558 | 0.771 - 1.618 | 1.281 | 0.206 | 1.540 | 0.123 | 0.935 - 1.756 |
| Anxiety Symptoms | 0.749 | 0.323 | -0.671 | 0.502 | 0.322 - 1.743 | 0.942 | 0.247 | -0.229 | 0.819 | 0.563 - 1.574 |
| Depression Symptoms | 1.657 | 0.756 | 1.107 | 0.268 | 0.678 - 4.050 | 1.935* | 0.528 | 2.419 | 0.016 | 1.133 - 3.303 |
| Poor Self-rated Health Status | 1.130 | 0.223 | 0.620 | 0.535 | 0.767 - 1.665 | 1.368 | 0.259 | 1.655 | 0.098 | 0.944 - 1.984 |
| School Bullying Victim (No=0) | 1.131 | 0.481 | 0.289 | 0.773 | 0.491 - 2.602 | 1.356 | 0.585 | 0.707 | 0.479 | 0.583 - 3.158 |
| School Bullying Perpetrator (No=0) | 1.473 | 0.517 | 1.104 | 0.270 | 0.740 - 2.930 | 0.990 | 0.325 | -0.031 | 0.975 | 0.520 - 1.885 |
| Constant | 0.038 | 0.066 | -1.890 | 0.059 | 0.001 - 1.128 | 0.001*** | 0.002 | -5.204 | 0.000 | 0.000 - 0.017 |
| Observations | 546 | | | | | 797 | | | | |
| DF | 11 | | | | | 11 | | | | |
| Chi^2^ | 16.42 | | | | | 52.98 | | | | |
| Log likelihood | -142.3 | | | | | -224.4 | | | | |

*** p<0.001, ** p<0.01, * p<0.05

**Table S11: Risk Factors and Association with Subjective Binging in Bisexual School Adolescents**

|  | Male adolescents | | | | | Female adolescents | | | | |
| --- | --- | --- | --- | --- | --- | --- | --- | --- | --- | --- |
|  | Odds Ratio | Std. Err. | *Z* value | *P* value | [95% Conf. Interval] | Odds Ratio | Std. Err. | *Z* value | *P* value | [95% Conf. Interval] |
| Age | 1.176 | 0.099 | 1.919 | 0.055 | 0.997 - 1.388 | 1.104 | 0.079 | 1.378 | 0.168 | 0.959 - 1.270 |
| Household Registration Residence (Urban=0) | 0.611 | 0.154 | -1.948 | 0.051 | 0.372 - 1.003 | 1.139 | 0.253 | 0.586 | 0.558 | 0.737 - 1.759 |
| Being the Only Child (No=0) | 0.760 | 0.186 | -1.119 | 0.263 | 0.470 - 1.229 | 1.037 | 0.243 | 0.155 | 0.877 | 0.655 - 1.642 |
| Suicidal Ideation (No=0) | 1.940* | 0.595 | 2.160 | 0.031 | 1.063 - 3.539 | 1.050 | 0.301 | 0.172 | 0.864 | 0.599 - 1.843 |
| Non-Suicidal Self-Harm (No=0) | 1.349 | 0.475 | 0.851 | 0.395 | 0.677 - 2.691 | 0.758 | 0.216 | -0.974 | 0.330 | 0.434 - 1.323 |
| Perceived Cognitive Deficits | 1.032 | 0.145 | 0.225 | 0.822 | 0.783 - 1.360 | 1.209 | 0.169 | 1.359 | 0.174 | 0.920 - 1.589 |
| Anxiety Symptoms | 1.781* | 0.514 | 1.999 | 0.046 | 1.011 - 3.135 | 0.575* | 0.137 | -2.326 | 0.020 | 0.361 - 0.917 |
| Depression Symptoms | 0.607 | 0.198 | -1.530 | 0.126 | 0.321 - 1.151 | 2.154** | 0.522 | 3.165 | 0.002 | 1.339 - 3.464 |
| Poor Self-rated Health Status | 1.070 | 0.154 | 0.466 | 0.641 | 0.806 - 1.420 | 1.174 | 0.184 | 1.024 | 0.306 | 0.864 - 1.597 |
| School Bullying Victim (No=0) | 0.806 | 0.274 | -0.634 | 0.526 | 0.414 - 1.571 | 0.952 | 0.381 | -0.123 | 0.902 | 0.435 - 2.084 |
| School Bullying Perpetrator (No=0) | 1.160 | 0.301 | 0.571 | 0.568 | 0.697 - 1.930 | 1.683* | 0.438 | 1.999 | 0.046 | 1.010 - 2.805 |
| Constant | 0.016*** | 0.019 | -3.447 | 0.001 | 0.001 - 0.166 | 0.014*** | 0.015 | -4.075 | 0.000 | 0.002 - 0.109 |
| Observations | 549 | | | | | 801 | | | | |
| DF | 11 | | | | | 11 | | | | |
| Chi^2^ | 21.66 | | | | | 32.43 | | | | |
| Log likelihood | -246.7 | | | | | -300.6 | | | | |

*** p<0.001, ** p<0.01, * p<0.05

**Table S12: Risk Factors and Association with Binge Eating in Bisexual School Adolescents**

|  | Male adolescents | | | | | Female adolescents | | | | |
| --- | --- | --- | --- | --- | --- | --- | --- | --- | --- | --- |
|  | Odds Ratio | Std. Err. | *Z* value | *P* value | [95% Conf. Interval] | Odds Ratio | Std. Err. | *Z* value | *P* value | [95% Conf. Interval] |
| Age | 1.089 | 0.081 | 1.142 | 0.254 | 0.941 - 1.259 | 1.042 | 0.060 | 0.723 | 0.470 | 0.932 - 1.166 |
| Household Registration Residence (Urban=0) | 0.670 | 0.145 | -1.852 | 0.064 | 0.439 - 1.024 | 1.201 | 0.205 | 1.072 | 0.284 | 0.859 - 1.679 |
| Being the Only Child (No=0) | 0.788 | 0.169 | -1.114 | 0.265 | 0.518 - 1.199 | 0.953 | 0.174 | -0.266 | 0.790 | 0.667 - 1.362 |
| Suicidal Ideation (No=0) | 1.526 | 0.418 | 1.543 | 0.123 | 0.892 - 2.611 | 1.072 | 0.232 | 0.320 | 0.749 | 0.701 - 1.638 |
| Non-Suicidal Self-Harm (No=0) | 1.947* | 0.605 | 2.146 | 0.032 | 1.059 - 3.580 | 0.986 | 0.212 | -0.063 | 0.950 | 0.647 - 1.504 |
| Perceived Cognitive Deficits | 1.336* | 0.165 | 2.338 | 0.019 | 1.048 - 1.702 | 1.638*** | 0.176 | 4.584 | 0.000 | 1.326 - 2.023 |
| Anxiety Symptoms | 2.137** | 0.546 | 2.974 | 0.003 | 1.295 - 3.525 | 0.913 | 0.160 | -0.523 | 0.601 | 0.648 - 1.286 |
| Depression Symptoms | 0.613 | 0.176 | -1.708 | 0.088 | 0.350 - 1.075 | 2.006*** | 0.373 | 3.744 | 0.000 | 1.393 - 2.887 |
| Poor Self-rated Health Status | 1.331* | 0.169 | 2.257 | 0.024 | 1.038 - 1.706 | 1.280* | 0.153 | 2.071 | 0.038 | 1.013 - 1.617 |
| School Bullying Victim (No=0) | 1.307 | 0.365 | 0.957 | 0.338 | 0.756 - 2.259 | 1.449 | 0.485 | 1.107 | 0.268 | 0.752 - 2.792 |
| School Bullying Perpetrator (No=0) | 1.425 | 0.318 | 1.589 | 0.112 | 0.921 - 2.205 | 1.515 | 0.328 | 1.920 | 0.055 | 0.991 - 2.315 |
| Constant | 0.031** | 0.033 | -3.261 | 0.001 | 0.004 - 0.249 | 0.036*** | 0.030 | -3.986 | 0.000 | 0.007 - 0.183 |
| Observations | 549 | | | | | 801 | | | | |
| DF | 11 | | | | | 11 | | | | |
| Chi^2^ | 69.17 | | | | | 142.1 | | | | |
| Log likelihood | -301 | | | | | -446.7 | | | | |

*** p<0.001, ** p<0.01, * p<0.05

**Table S13: Risk Factors and Association with Dietary Restriction in Reporting No Sexual Attraction School Adolescents**

|  | Male adolescents | | | | | Female adolescents | | | | |
| --- | --- | --- | --- | --- | --- | --- | --- | --- | --- | --- |
|  | Odds Ratio | Std. Err. | *Z* value | *P* value | [95% Conf. Interval] | Odds Ratio | Std. Err. | *Z* value | *P* value | [95% Conf. Interval] |
| Age | 0.978 | 0.044 | -0.492 | 0.623 | 0.896 - 1.068 | 1.036 | 0.037 | 0.980 | 0.327 | 0.966 - 1.111 |
| Household Registration Residence (Urban=0) | 0.907 | 0.090 | -0.990 | 0.322 | 0.747 - 1.101 | 0.986 | 0.093 | -0.150 | 0.881 | 0.820 - 1.186 |
| Being the Only Child (No=0) | 0.927 | 0.092 | -0.765 | 0.444 | 0.764 - 1.125 | 0.936 | 0.097 | -0.643 | 0.520 | 0.764 - 1.146 |
| Suicidal Ideation (No=0) | 1.102 | 0.205 | 0.522 | 0.602 | 0.765 - 1.586 | 1.448* | 0.224 | 2.394 | 0.017 | 1.069 - 1.960 |
| Non-Suicidal Self-Harm (No=0) | 0.949 | 0.188 | -0.263 | 0.792 | 0.643 - 1.401 | 1.861*** | 0.299 | 3.860 | 0.000 | 1.358 - 2.551 |
| Perceived Cognitive Deficits | 0.943 | 0.059 | -0.929 | 0.353 | 0.834 - 1.067 | 1.137* | 0.073 | 1.995 | 0.046 | 1.002 - 1.290 |
| Anxiety Symptoms | 0.887 | 0.111 | -0.959 | 0.337 | 0.695 - 1.133 | 1.143 | 0.127 | 1.201 | 0.230 | 0.919 - 1.422 |
| Depression Symptoms | 1.467** | 0.203 | 2.770 | 0.006 | 1.119 - 1.925 | 0.930 | 0.121 | -0.559 | 0.576 | 0.720 - 1.201 |
| Poor Self-rated Health Status | 1.445*** | 0.085 | 6.257 | 0.000 | 1.287 - 1.621 | 1.318*** | 0.082 | 4.466 | 0.000 | 1.168 - 1.488 |
| School Bullying Victim (No=0) | 1.245 | 0.215 | 1.269 | 0.204 | 0.887 - 1.747 | 1.116 | 0.242 | 0.506 | 0.613 | 0.729 - 1.708 |
| School Bullying Perpetrator (No=0) | 1.147 | 0.154 | 1.021 | 0.307 | 0.882 - 1.492 | 1.053 | 0.189 | 0.285 | 0.776 | 0.740 - 1.498 |
| Constant | 0.282* | 0.177 | -2.016 | 0.044 | 0.083 - 0.965 | 0.176*** | 0.090 | -3.407 | 0.001 | 0.065 - 0.478 |
| Observations | 2,215 | | | | | 2,101 | | | | |
| DF | 11 | | | | | 11 | | | | |
| Chi^2^ | 80.16 | | | | | 104.2 | | | | |
| Log likelihood | -1330 | | | | | -1377 | | | | |

*** p<0.001, ** p<0.01, * p<0.05**Table S14: Risk Factors and Association with Purging in Reporting No Sexual Attraction School Adolescents**

|  | Male adolescents | | | | | Female adolescents | | | | |
| --- | --- | --- | --- | --- | --- | --- | --- | --- | --- | --- |
|  | Odds Ratio | Std. Err. | *Z* value | *P* value | [95% Conf. Interval] | Odds Ratio | Std. Err. | *Z* value | *P* value | [95% Conf. Interval] |
| Age | 0.961 | 0.096 | -0.398 | 0.690 | 0.790 - 1.169 | 1.112 | 0.095 | 1.249 | 0.212 | 0.941 - 1.315 |
| Household Registration Residence (Urban=0) | 1.131 | 0.247 | 0.564 | 0.573 | 0.738 - 1.734 | 1.323 | 0.312 | 1.189 | 0.234 | 0.834 - 2.100 |
| Being the Only Child (No=0) | 0.645 | 0.156 | -1.815 | 0.070 | 0.402 - 1.036 | 1.228 | 0.312 | 0.807 | 0.420 | 0.746 - 2.020 |
| Suicidal Ideation (No=0) | 0.979 | 0.351 | -0.060 | 0.952 | 0.485 - 1.976 | 1.301 | 0.405 | 0.845 | 0.398 | 0.707 - 2.395 |
| Non-Suicidal Self-Harm (No=0) | 1.424 | 0.506 | 0.996 | 0.319 | 0.710 - 2.856 | 2.165** | 0.637 | 2.626 | 0.009 | 1.216 - 3.853 |
| Perceived Cognitive Deficits | 1.053 | 0.138 | 0.393 | 0.695 | 0.815 - 1.360 | 0.923 | 0.142 | -0.517 | 0.605 | 0.682 - 1.249 |
| Anxiety Symptoms | 1.025 | 0.256 | 0.098 | 0.922 | 0.628 - 1.673 | 1.503 | 0.378 | 1.620 | 0.105 | 0.918 - 2.461 |
| Depression Symptoms | 1.476 | 0.394 | 1.459 | 0.145 | 0.875 - 2.491 | 1.183 | 0.339 | 0.586 | 0.558 | 0.675 - 2.073 |
| Poor Self-rated Health Status | 1.430** | 0.183 | 2.796 | 0.005 | 1.113 - 1.838 | 1.342 | 0.212 | 1.865 | 0.062 | 0.985 - 1.828 |
| School Bullying Victim (No=0) | 1.207 | 0.403 | 0.564 | 0.573 | 0.628 - 2.321 | 1.492 | 0.567 | 1.051 | 0.293 | 0.708 - 3.144 |
| School Bullying Perpetrator (No=0) | 1.355 | 0.357 | 1.153 | 0.249 | 0.808 - 2.272 | 1.259 | 0.462 | 0.627 | 0.531 | 0.613 - 2.584 |
| Constant | 0.027* | 0.038 | -2.564 | 0.010 | 0.002 - 0.427 | 0.002*** | 0.002 | -4.950 | 0.000 | 0.000 - 0.023 |
| Observations | 2,209 | | | | | 2,097 | | | | |
| DF | 11 | | | | | 11 | | | | |
| Chi^2^ | 40.43 | | | | | 58.25 | | | | |
| Log likelihood | -374.7 | | | | | -317.1 | | | | |

*** p<0.001, ** p<0.01, * p<0.05

**Table S15: Risk Factors and Association with Subjective Binging in Reporting No Sexual Attraction School Adolescents**

|  | Male adolescents | | | | | Female adolescents | | | | |
| --- | --- | --- | --- | --- | --- | --- | --- | --- | --- | --- |
|  | Odds Ratio | Std. Err. | *Z* value | *P* value | [95% Conf. Interval] | Odds Ratio | Std. Err. | *Z* value | *P* value | [95% Conf. Interval] |
| Age | 1.156* | 0.070 | 2.401 | 0.016 | 1.027 - 1.300 | 1.122 | 0.069 | 1.882 | 0.060 | 0.995 - 1.265 |
| Household Registration Residence (Urban=0) | 0.958 | 0.138 | -0.295 | 0.768 | 0.723 - 1.270 | 0.986 | 0.171 | -0.078 | 0.938 | 0.702 - 1.387 |
| Being the Only Child (No=0) | 0.938 | 0.137 | -0.440 | 0.660 | 0.705 - 1.248 | 0.992 | 0.189 | -0.044 | 0.965 | 0.682 - 1.441 |
| Suicidal Ideation (No=0) | 1.180 | 0.291 | 0.670 | 0.503 | 0.728 - 1.912 | 0.805 | 0.215 | -0.813 | 0.416 | 0.476 - 1.359 |
| Non-Suicidal Self-Harm (No=0) | 1.271 | 0.329 | 0.926 | 0.354 | 0.765 - 2.109 | 0.632 | 0.184 | -1.580 | 0.114 | 0.357 - 1.117 |
| Perceived Cognitive Deficits | 1.274** | 0.108 | 2.865 | 0.004 | 1.080 - 1.504 | 1.444*** | 0.159 | 3.331 | 0.001 | 1.163 - 1.793 |
| Anxiety Symptoms | 1.473* | 0.248 | 2.298 | 0.022 | 1.059 - 2.050 | 0.905 | 0.172 | -0.523 | 0.601 | 0.624 - 1.314 |
| Depression Symptoms | 0.802 | 0.152 | -1.166 | 0.244 | 0.554 - 1.162 | 1.684* | 0.362 | 2.427 | 0.015 | 1.106 - 2.566 |
| Poor Self-rated Health Status | 0.964 | 0.082 | -0.430 | 0.667 | 0.816 - 1.139 | 0.984 | 0.112 | -0.138 | 0.890 | 0.788 - 1.230 |
| School Bullying Victim (No=0) | 1.221 | 0.279 | 0.873 | 0.382 | 0.780 - 1.911 | 1.067 | 0.375 | 0.186 | 0.853 | 0.536 - 2.127 |
| School Bullying Perpetrator (No=0) | 1.843*** | 0.313 | 3.595 | 0.000 | 1.320 - 2.572 | 1.419 | 0.404 | 1.227 | 0.220 | 0.811 - 2.480 |
| Constant | 0.010*** | 0.009 | -5.386 | 0.000 | 0.002 - 0.054 | 0.008*** | 0.007 | -5.459 | 0.000 | 0.001 - 0.045 |
| Observations | 2,215 | | | | | 2,105 | | | | |
| DF | 11 | | | | | 11 | | | | |
| Chi^2^ | 59.17 | | | | | 49.41 | | | | |
| Log likelihood | -747 | | | | | -536.3 | | | | |

*** p<0.001, ** p<0.01, * p<0.05

**Table S16: Risk Factors and Association with Binge Eating in Reporting No Sexual Attraction School Adolescents**

|  | Male adolescents | | | | | Female adolescents | | | | |
| --- | --- | --- | --- | --- | --- | --- | --- | --- | --- | --- |
|  | Odds Ratio | Std. Err. | *Z* value | *P* value | [95% Conf. Interval] | Odds Ratio | Std. Err. | *Z* value | *P* value | [95% Conf. Interval] |
| Age | 1.054 | 0.054 | 1.028 | 0.304 | 0.954 - 1.165 | 1.094* | 0.049 | 2.034 | 0.042 | 1.003 - 1.194 |
| Household Registration Residence (Urban=0) | 0.994 | 0.115 | -0.050 | 0.960 | 0.793 - 1.247 | 0.910 | 0.112 | -0.764 | 0.445 | 0.715 - 1.158 |
| Being the Only Child (No=0) | 0.819 | 0.097 | -1.684 | 0.092 | 0.649 - 1.033 | 1.093 | 0.145 | 0.668 | 0.504 | 0.842 - 1.419 |
| Suicidal Ideation (No=0) | 1.136 | 0.227 | 0.638 | 0.524 | 0.768 - 1.680 | 1.079 | 0.190 | 0.430 | 0.667 | 0.764 - 1.523 |
| Non-Suicidal Self-Harm (No=0) | 1.304 | 0.274 | 1.260 | 0.208 | 0.863 - 1.970 | 1.279 | 0.229 | 1.378 | 0.168 | 0.901 - 1.816 |
| Perceived Cognitive Deficits | 1.432*** | 0.098 | 5.259 | 0.000 | 1.253 - 1.637 | 1.747*** | 0.137 | 7.091 | 0.000 | 1.497 - 2.038 |
| Anxiety Symptoms | 1.171 | 0.160 | 1.157 | 0.247 | 0.896 - 1.530 | 1.039 | 0.139 | 0.288 | 0.774 | 0.800 - 1.350 |
| Depression Symptoms | 1.360* | 0.203 | 2.055 | 0.040 | 1.014 - 1.823 | 1.732*** | 0.264 | 3.606 | 0.000 | 1.285 - 2.335 |
| Poor Self-rated Health Status | 1.053 | 0.072 | 0.758 | 0.448 | 0.921 - 1.204 | 1.151 | 0.092 | 1.769 | 0.077 | 0.985 - 1.345 |
| School Bullying Victim (No=0) | 1.121 | 0.215 | 0.598 | 0.550 | 0.771 - 1.632 | 1.364 | 0.323 | 1.312 | 0.189 | 0.858 - 2.168 |
| School Bullying Perpetrator (No=0) | 1.904*** | 0.268 | 4.568 | 0.000 | 1.444 - 2.509 | 1.812** | 0.359 | 3.002 | 0.003 | 1.229 - 2.671 |
| Constant | 0.051*** | 0.037 | -4.142 | 0.000 | 0.012 - 0.209 | 0.014*** | 0.009 | -6.632 | 0.000 | 0.004 - 0.049 |
| Observations | 2,215 | | | | | 2,105 | | | | |
| DF | 11 | | | | | 11 | | | | |
| Chi^2^ | 166.6 | | | | | 268.1 | | | | |
| Log likelihood | -1032 | | | | | -912.3 | | | | |

*** p<0.001, ** p<0.01, * p<0.05
